# Artemisinic acid attenuates osteoclast formation and titanium particle-induced osteolysis via inhibition of RANKL-induced ROS accumulation and MAPK and NF-κB signaling pathways

**DOI:** 10.3389/fphar.2024.1345380

**Published:** 2024-05-01

**Authors:** Tian Gao, Chaohong Yu, Xiaofeng Shi, Yuehao Hu, Yongyun Chang, Jingwei Zhang, Yitian Wang, Zanjing Zhai, Xinlin Jia, Yuanqing Mao

**Affiliations:** Shanghai Key Laboratory of Orthopedic Implants, Department of Orthopedic Surgery, Shanghai Ninth People’s Hospital, Shanghai Jiaotong University School of Medicine, Shanghai, China

**Keywords:** artemisinic acid, osteoclastogenesis, reactive oxygen species, NF-κB, MAPK, osteolysis

## Abstract

Periprosthetic osteolysis (PPO) is the most common cause of joint arthroplasty failure. Its progression involves both biological and mechanical factors. Osteoclastogenesis induced by wear from debris-cell interactions, ultimately leading to excessive bone erosion, is considered the primary cause of PPO; therefore, targeting osteoclasts is a promising treatment approach. Currently available drugs have various side effects and limitations. Artemisinic acid (ArA) is a sesquiterpene isolated from the traditional herb *Artemisia annua* L. that has various pharmacological effects, such as antimalarial, anti-inflammatory, and antioxidant activities. Therefore, this study was aimed at investigating the effect of ArA on osteoclast formation and bone resorption function *in vitro*, as well as wear particle-induced osteolysis *in vivo*, and to explore its molecular mechanism of action. Here, we report that ArA inhibits RANKL-stimulated osteoclast formation and function. Mechanistically, ArA suppresses intracellular reactive oxygen species levels by activating the antioxidant response via nuclear factor erythroid-2-related factor 2 (Nrf2) pathway upregulation. It also inhibits the mitogen-activated kinases (MAPK) and nuclear factor-κB (NF-κB) pathways, as well as the transcription and expression of NFATc1 and c-Fos. *In vivo* experiments demonstrated that ArA reduces osteoclast formation and alleviates titanium particle-induced calvarial osteolysis. Collectively, our study highlights that ArA, with its osteoprotective and antioxidant effects, is a promising therapeutic agent for preventing and treating PPO and other osteoclast-mediated osteolytic diseases.

## 1 Introduction

Total joint arthroplasty (TJA) is a common and effective treatment for severe late-stage destructive joint diseases, including arthritis, osteonecrosis, and osteoporotic fractures. TJA greatly improves patients’ quality of life (Learmonth et al., 2007; Wilson et al., 2019; Madry, 2022). Periprosthetic osteolysis (PPO) and aseptic loosening (AL) have been identified as the main culprits behind TJA surgery failure, occurring in up to 25% of TJA patients (Bozic et al., 2009; Girard et al., 2013; Sadoghi et al., 2013; Patel et al., 2015). These complications have a significant negative impact on patient prognosis and financial burden (Bozic et al., 2009). Many studies have confirmed that in the absence of infection and dislocation, the accumulation of wear debris generated by the biomechanics of prosthetic materials, such as titanium, chromium, and polyethylene, at the bone-implant contact surface triggers the development of PPO and AL (Gallo et al., 2014; Goodman and Gallo, 2019; Mcarthur et al., 2019; Goodman et al., 2022). Macrophages are then recruited to the area, where they phagocytose wear debris and secrete pro-inflammatory factors, along with T lymphocytes, fibroblasts, and foreign body giant cells (Goodman, 2007; Girard et al., 2013). These pro-inflammatory factors, including interleukin 1β, tumor necrosis factor-α, interleukin 6, and prostaglandin E2, stimulate osteoblasts and marrow stromal cells to oversynthesize Receptor Activator of Nuclear Factor-κ B Ligand (RANKL) (Boyle et al., 2003; Holding et al., 2006). High levels of RANKL at the prosthetic site bind with the highly expressed receptor activator for nuclear factor-κ (RANK) on the progenitor cells of osteoclasts and recruit tumor necrosis factor receptor-associated factor 6 (TRAF6) (Gohda et al., 2005). This upregulates the MAPK signaling cascade, which includes p38 MAPK, c-Jun N-terminal kinase (JNK), and extracellular signal-regulated kinase (ERK), and ultimately activates NFATc1 and c-Fos (Suda et al., 2001; Holding et al., 2006), thereby causing an inflammatory response (Purdue et al., 2007) and excessive osteoclastogenesis. Excessive osteoclastogenesis and bone resorption are generally considered the primary pathologies of PPO (Eger et al., 2018). Moreover, wear particles cause excessive reactive oxygen species (ROS), which promote the proliferation of osteoclast progenitor cells and the M1 polarization of macrophages, thereby producing more ROS and inflammatory factors (Sun et al., 2021), aggravating osteoclastogenesis, and triggering bone resorption (Ha et al., 2004; Lee et al., 2005; Agidigbi and Kim, 2019). Current research on PPO treatment has two main directions: one is to improve surgical techniques and implant design, searching for more biocompatible materials to manufacture prostheses. However, completely avoiding the production of wear particles by the implant is likely to be unfeasible (Sundfeldt et al., 2006; Holt et al., 2007). The second is to identify compounds that inhibit wear particle-induced macrophage inflammatory responses, osteoclast differentiation, and bone resorption. Osteoclasts are confirmed to play a major role in inflammatory osteolysis and subsequent PPO and AL (Hotokezaka et al., 2007). Therefore, identifying substances that suppress intracellular ROS levels and osteoclastogenesis represents a promising approach to ameliorating osteoclast-mediated osteolytic diseases such as PPO, arthritis, and postmenopausal osteoporosis.

Several drugs, including denosumab and odanacatib, have been developed to inhibit osteoclastogenesis (Kendler, 2011; Rachner et al., 2011; Reid and Billington, 2022). However, these drugs are not suitable for long-term treatment due to their various side effects, such as cardiovascular disorders (Dai et al., 2020), spinal fractures, and femoral head necrosis (Reid and Billington, 2022). Recently, some natural herbs and their active ingredients have been identified as having potential inhibitory effects on the MAPK, NF-κB, PI3K/Akt, and other osteoclast-related signaling pathways (Nam, 2006; Deng et al., 2020). This coincides with the traditional Chinese medical use of herbs to treat various diseases (Katiyar et al., 2012; Teschke et al., 2015; Li et al., 2021). Among these herbs, *Artemisia annua* L. has been used to treat malaria for several centuries. It also has antioxidant, anticancer, antibacterial, and anti-inflammatory activities (Chaturvedi et al., 2010; Bilia et al., 2014; Ho et al., 2014; Guo et al., 2022) due to the presence of sesquiterpenes, flavonoids, and essential oils, including the sesquiterpene artemisinic acid (ArA). Similar to its source material, ArA exhibits various pharmacological activities, including antimalarial (Chaturvedi et al., 2010) and antioxidant activities (Ferreira and Luthria, 2010). However, its effects on bone resorption, osteoclast formation, and intracellular ROS production remain unknown.

Therefore, the present study aimed to elucidate the effects of ArA on osteoclast formation and function, as well as ROS accumulation, during osteoclast formation. We hypothesized that ArA has an osteoprotective effect on Ti particle-induced osteolysis and inhibits RANKL-induced osteoclast formation and bone resorption. The primary endpoints were to evaluate 1) changes in mouse calvarial bone mineral density (BMD) and TRAP^+^ osteoclast abundance after ArA treatment *in vivo* and 2) changes in RANKL-induced osteoclast abundance and area after ArA treatment *in vitro*. This study reports the bone-protective effect of ArA, a cost-effective agent with good biological safety, broadens the application prospects of small molecules derived from Chinese herbal medicine, and provides new ideas for the clinical prevention and treatment of PPO.

## 2 Materials and methods

### 2.1 Media and reagents

Artemisic acid (C_15_H_22_O_2,_ purity ≥99.73%) was obtained from MedChemExpress (Shanghai, China). Macrophage colony-stimulating factor (M-CSF) and recombinant mouse RANKL were purchased from R&D Systems (Minneapolis, MN, United States). Phosphate-buffered saline (PBS), Eagle’s minimum essential medium, alpha modification medium (α-MEM), and penicillin/streptomycin were purchased from Gibco BRL (Gaithersburg, MD, United States). Fetal bovine serum (FBS) was obtained from Avantor (Radnor, PA, United States). The Cell Counting Kit-8 (CCK-8) was obtained from Beyotime Biotechnology Inc. (Shanghai, China). TRIzol reagent was purchased from Invitrogen (Carlsbad, CA, United States). The Prime Script RT reagent kit and TB Green™ Premix Ex Taq™ II were obtained from Takara Biomedical Technology (Beijing, China). DAPI, the Actin-Tracker Red-Rhodamine, and DCFH-DA probes were obtained from Beyotime Biotechnology Inc. (Shanghai, China). Primary antibodies targeting p-p65, p-ERK, p-JNK, p-p38, total ERK, total JNK, total p38, and total p65 were purchased from Cell Signaling Technology, Inc. (Beverly, MA, United States). NFATc1, c-fos, IκBα, nrf2, p-nrf2, NQO1, and β-actin antibodies were purchased from Affinity (San Jose, CA, United States). Ti particles (<20 μm diameter) were purchased from Alfa Aesar (Ward Hill, MA, United States). To remove possible adherent endotoxins, the particles were baked for 8 h at 180°C and then washed in 75% ethanol for 48 h. Subsequently, Ti particles were resuspended in sterile PBS at a concentration of 100 mg/mL using an LP vortex mixer (ThermoFisher Scientific, Scoresby, Australia) and stored at 4°C until further use.

### 2.2 Cell culture

We obtained primary BMMs from 6-week-old C57BL/6J mice by irrigating the marrow cavities of the femur and tibia with a sterile syringe (Toro et al., 2012; Li et al., 2013). Cells were cultured in α-MEM complete medium, which is α-MEM with 10% (v/v) FBS and 1% (w/v) penicillin/streptomycin) in a volume containing 30 ng/mL M-CSF in a 5% CO_2_ humidified incubator at 37°C for the first 3–5 days. The medium was changed every 2–3 days until the cells reached approximately 90% confluency in the culture dish. After dissociation with trypsin, the cells were transferred to culture plates or dishes for subsequent studies. A schematic design regarding different *in vitro* incubation times is displayed in Supplementary Figure S1.

### 2.3 Cell viability assay

The cytotoxic effects of ArA on BMM cells were investigated using a CCK-8 assay. Briefly, BMMs (8 × 10^3^ cells/well) were transferred to 96-well plates in triplicate and incubated for 24 h in α-MEM complete medium containing 30 ng/mL M-CSF. Cells were then treated with different concentrations of ArA (0, 2.5, 5, 10, 20, 40, 60, 80, and 160 μM) for 72 h, and the culture medium changed after the first 48 h. The CCK-8 assay was performed according to the manufacturer’s instructions. Culture medium (100 μL) containing 10% CCK-8 regent (v/v) was added to each well and incubated at 37°C for 2 h. Absorbance at 450 nm was measured using an Infinite M200 Pro microplate reader (Tecan Life Sciences, Männedorf, Switzerland).

### 2.4 Detection of intracellular ROS

Intracellular ROS were detected using a DCFH-DA probe, according to the manufacturer’s instructions. Briefly, BMMs were seeded into confocal dishes (Cellvis, Mountain View, CA, United States) and cultured with or without 100 ng/mL RANKL, 30 ng/mL M-CSF, and different concentrations of ArA for 24 h. DCFH-DA was diluted 1:1,000 with serum-free medium to a final concentration of 10 μmol/L. The cells were incubated with this medium at 37°C for 20 min and then washed three times with serum-free cell culture medium to remove the uncombined probes. Fluorescence images were captured using a DM4000B epifluorescence microscope (Leica Microsystems, Wetzlar, Germany). Semi-quantitative analysis of ROS-positive cells was performed using ImageJ 1.53t (NIH, Bethesda, MD, United States).

### 2.5 Osteoclastogenesis assay *in vitro*

Osteoclast formation was visualized with the tartrate-resistant acid phosphatase (TRAP) regent. BMMs were seeded in 96-well plates in triplicate (8 × 10^3^ cells/well) for 24 h before α-MEM complete medium was removed from each well. Then, based on the cell viability results, BMMs were incubated with varying non-cytotoxic concentrations of ArA (0, 20, 40, and 80 µM), M-CSF (30 ng/mL), and RANKL (100 ng/mL). The culture medium was changed every 2 days. After approximately 6–8 days, the mature osteoclasts were observed and were subsequently fixed with 4% paraformaldehyde and stained with TRAP regent at 37°C for 1 h. The quantity and surface of TRAP^+^ multinucleated cells were calculated using ImageJ 1.53t (NIH, Bethesda, MD, United States).

### 2.6 Bone resorption assay

Bone resorption was observed through bone slice resorption pits. Bovine bone slices (JoyTech Bio Co., Ltd., Zhejiang, China) were sterilized in 70% ethanol for 2 h, then soaked in PBS overnight to remove the alcohol, and then soaked in α-MEM for 4 h before use. BMMs were transferred in groups of three to 96-well plates with pre-positioned, prepared bone slices. After incubation for 24 h, the osteoclasts were incubated with RANKL (100 ng/mL), M-CSF (30 ng/mL), and different concentrations of ArA (0, 20, 40, and 80 μM) for 8 days. The cells were then removed from the bone slices with sodium hypochlorite. After washing three times with PBS, bone slices were photographed using a scanning electron microscope (Zeiss Sigma 500/Oxford EDS). The area of the resorption pits was determined using ImageJ 1.53t (NIH, Bethesda, MD).

### 2.7 F-actin ring formation assay

F-actin was visualized by immunofluorescence. BMMs (2 × 10^4^ cells/dish) were seeded in confocal dishes and incubated with M-CSF (30 ng/mL), RANKL (100 ng/mL), and different concentrations of ArA (0, 20, 40, and 80 μM). After the mature osteoclasts were observed, the cells were fixed with 4% paraformaldehyde for 10 min, followed by permeabilization with PBS containing 0.1% Triton X-100 (v/v) (Sigma-Aldrich) for 5 min. F-actin rings were stained with Actin-Tracker Red-Rhodamine, and cell nuclei were stained with DAPI. Actin rings were visualized with a DM4000B epifluorescence microscope (Leica Microsystems, Wetzlar, Germany), and ImageJ 1.53t (NIH, Bethesda, MD) was used to analyze the images.

### 2.8 RNA extraction and reverse transcription quantitative polymerase chain reaction (RT-qPCR)

The transcript levels of osteoclast-related genes were detected by RT-qPCR. BMMs (2 × 10^5^ cells/well) were seeded into 6-well plates supplemented with M-CSF (30 ng/mL), RANKL (100 ng/mL), and different concentrations of ArA (0, 10, 20, 40, and 80 μM) for 3 days. The TRIzol reagent was used to lyse the cells and extract total RNA. The entire experimental process of RT-qPCR strictly followed the MIQE guidelines (Taylor et al., 2010). RNA concentration and purity were determined using a Nanodrop 2000 instrument (Thermo Fisher Scientific, Waltham, MA, United States), ensuring that OD260/280 was between 1.8 and 2.0. Equal amounts of RNA (1,000 ng) were then reverse transcribed to synthesize complementary DNA (cDNA) to avoid inflating the differences between groups. According to the manufacturer instructions for TB Green™ Premix Ex Taq™ II, a 10 μL reaction system was established and used for the RT-qPCR assay on an ABI 7500 Sequencing Detection System (Thermo Fisher Scientific). Cycling conditions were set to 40 cycles (95°C for 5 s and 60°C for 30 s). Melting curves were examined to verify amplification specificity. Relative gene expression was calculated using the comparative 2^−ΔΔCT^ method (Livak and Schmittgen, 2001). The primer sequences were as shown in Table 1, among which *Gapdh* was used as the housekeeping gene.

**TABLE 1:** The specific primers used in quantitative real-time PCR (qRT-PCR).

Target gene	Primer Sequence (5′-3′)	
	Forward	Reverse
*Ctsk*	CTT​CCA​ATA​CGT​GCA​GCA​GA	TCT​TCA​GGG​CTT​TCT​CGT​TC
*Trap*	CTG​GAG​TGC​ACG​ATG​CCA​GCG​ACA	TCC​GTG​CTC​GGC​GAT​GGA​CCA​GA
*Dcstamp*	AAA​ACC​CTT​GGG​CTG​TTC​TT	AAT​CAT​GGA​CGA​CTC​CTT​GG
*Ctr*	TGC​AGA​CAA​CTC​TTG​GTT​GG	TCG​GTT​TCT​TCT​CCT​CTG​GA
*c-Fos*	CCA​GTC​AAG​AGC​ATC​AGC​AA	AAG​TAG​TGC​AGC​CCG​GAG​TA
*Nfatc1*	CCG​TTG​CTT​CCA​GAA​AAT​AAC​A	TGT​GGG​ATG​TGA​ACT​CGG​AA
*Gapdh*	ACC​ACA​GTC​CAA​GCC​ATC​AC	CAC​ATT​GGG​GGT​AGG​AAC​AC

### 2.9 Osteoblastogenesis assay

Osteoblastogenesis was visualized by alkaline phosphatase (ALP) and alizarin red staining of bone mesenchymal stem cells (BMSCs). BMSCs were obtained from marrow cavities of the femur and tibia in 4–6-week-old male C57BL/6 mice, which were cultured with a complete medium consisting of α-MEM supplemented with 10% (v/v) FBS and 1% (w/v) penicillin/streptomycin. BMSCs (10 × 10^4^ cells/well) were seeded in 24-well plates and cultured with osteogenic medium (50 μg/mL ascorbic acid, 5 mM glycerol phosphate) with or without different concentrations of ArA. ALP and alizarin red staining were respectively performed on the 7th and 21st days of osteogenic differentiation induction, and the culture medium was changed every 2 days.

### 2.10 Western blotting analysis

Protein expression levels during osteoclast formation were detected using Western blotting. To detect the effect of ArA on the Nrf2, MAPK, and NF-κB signaling pathways, BMMs were seeded into 6-well plates at a density of 3 × 10^5^ cells/well with M-CSF (30 ng/mL) and different concentrations of ArA for 24 h. BMMs were then incubated with RANKL (100 ng/mL) for 25 min. To explore the time-dependent effects of ArA on NFATc1 and c-fos, the BMMs were incubated with ArA (40 µM), RANKL (100 ng/mL), and M-CSF (30 ng/mL) for different time periods. To determine the concentration-dependent effects of ArA on NFATc1 and c-Fos, cells were incubated with RANKL (100 ng/mL), M-CSF (30 ng/mL), and various doses of ArA for 4 days. The cells were washed with PBS and lysed with RIPA buffer with a protease and phosphatase inhibitor cocktail (Beyotime Biotechnology Inc.). After centrifugation, the collected proteins were dissolved in loading buffer, separated by 10% SDS-PAGE, and then transferred to 0.22 μm polyvinylidene fluoride membranes. The membranes were blocked with 1×TBST (Tris-buffered saline with Tween 20) containing 5% BSA on a shaker for 1 h at room temperature and were incubated with the relevant primary antibodies overnight at 4°C. After washing three times with TBST, the membranes were incubated with secondary antibodies for 70 min at room temperature. Blot detection was performed using an Odyssey V3.0 image scanning system (Li-COR Inc., Lincoln, NE, United States). Blotting results were analyzed and quantified using ImageJ 1.53t (NIH, Bethesda, MD, United States).

### 2.11 Ti particle-induced calvarial osteolysis mouse model

The animal protocol was approved by the Animal Care and Experiment Committee of the Ninth People’s Hospital Affiliated with the Shanghai Jiao Tong University School of Medicine (SH9H-2023-A862-1). All experiments were conducted in accordance with the American Psychological Association’s guidelines for Ethical Conduct in the Care and Use of Nonhuman Animals in Research. Based on previous reports (Liao et al., 2019; Liao et al., 2021; Wu et al., 2022), a Ti particle-induced mouse calvarial osteolysis model was used to evaluate the *in vivo* osteoprotective effect of ArA. Twenty 6-week-old male C57BL/6J mice (Jihui Laboratory Animal Breeding Co., Ltd., Shanghai, China), approximately weighing 22 ± 2 g, were randomly assigned to four different treatment groups with five mice each: 1) sham group (with PBS injection); 2) vehicle group (with 25 mg of Ti particles on the calvarial surface); 3) low-ArA group (with 25 mg of Ti particles on the calvarial surface and 5 mg/kg/d ArA in the abdominal cavity); 4) high-ArA group (with 25 mg of Ti particles on the calvarial surface and 10 mg/kg/d ArA intraperitoneally). To reduce the risk of ArA toxicity in mice, the doses used were determined by previous reports (Lee et al., 2017; Zhang, 2020) and our pilot study. Briefly, under 1.4% isoflurane general anesthesia, the mouse periosteum was separated from the calvaria, and a collagen sponge soaked in sterile PBS or titanium particle PBS solution was embedded under the periosteum at the midline of the calvaria. On the day after implantation, PBS with or without ArA (5 or 10 mg/kg/d) was injected intraperitoneally for 14 days. After this treatment, the mice were sacrificed, and the entire calvariae were dissected and washed with PBS. After removing the titanium particles, the calvariae were fixed by soaking in 4% PFA for 48 h for subsequent analysis.

### 2.12 Micro-CT scanning

High-resolution μCT-100 micro-CT (SCANCO Medical AG, Brüttisellen, Switzerland) with an isometric resolution of 10 μm was used to analyze the fixed calvarias. Microstructural indicators of bone volume/tissue volume (BV/TV) and bone mineral density (BMD) were determined in the regions of interest using supporting software (version 6.5-3, SCANCO Medical). Both indices are key morphometric parameters of bone mass.

### 2.13 Histological and immunohistochemical analysis

After micro-CT scanning, the calvaria samples were decalcified using 10% EDTA (pH = 7.4) for 14 days, embedded in paraffin, and prepared as histological sections before being stained with hematoxylin and eosin (H&E) and TRAP. Immunofluorescence staining was performed with antibodies against NFATc1 and Nrf2 (Affinity, San Jose, CA, United States). For the *in vivo* biosafety of ArA, the major organs of the mice, including the heart, liver, and kidneys, were collected at the same time when sacrificed 14 days after treatment and subsequently H&E stained. Images of stained sections were acquired with a Panoramic MIDI Scanner (3DHistech, Budapest, Hungary). The osteoclast/bone surface ratio (N.Oc/BS) and the osteoclast surface/bone surface ratio (OcS/BS) were calculated using ImageJ 1.53t.

### 2.14 Statistical analysis

GraphPad Prism v9.3.1 (GraphPad Software Inc., San Diego, CA, United States) was used for statistical analysis of the results. The data were all represented as the mean ± standard deviation (SD) of three or more independent experiments. The data were first tested for homogeneity of variance and then subjected to one-way analysis of variance (ANOVA), followed by Tukey’s *post hoc* analysis. Significant differences were determined at *p < 0.05, **p < 0.01, and ***p < 0.001.

## 3 Results

### 3.1 ArA dose-dependently suppressed RANKL-induced osteoclast formation at non-cytotoxic doses

**FIGURE 1:**
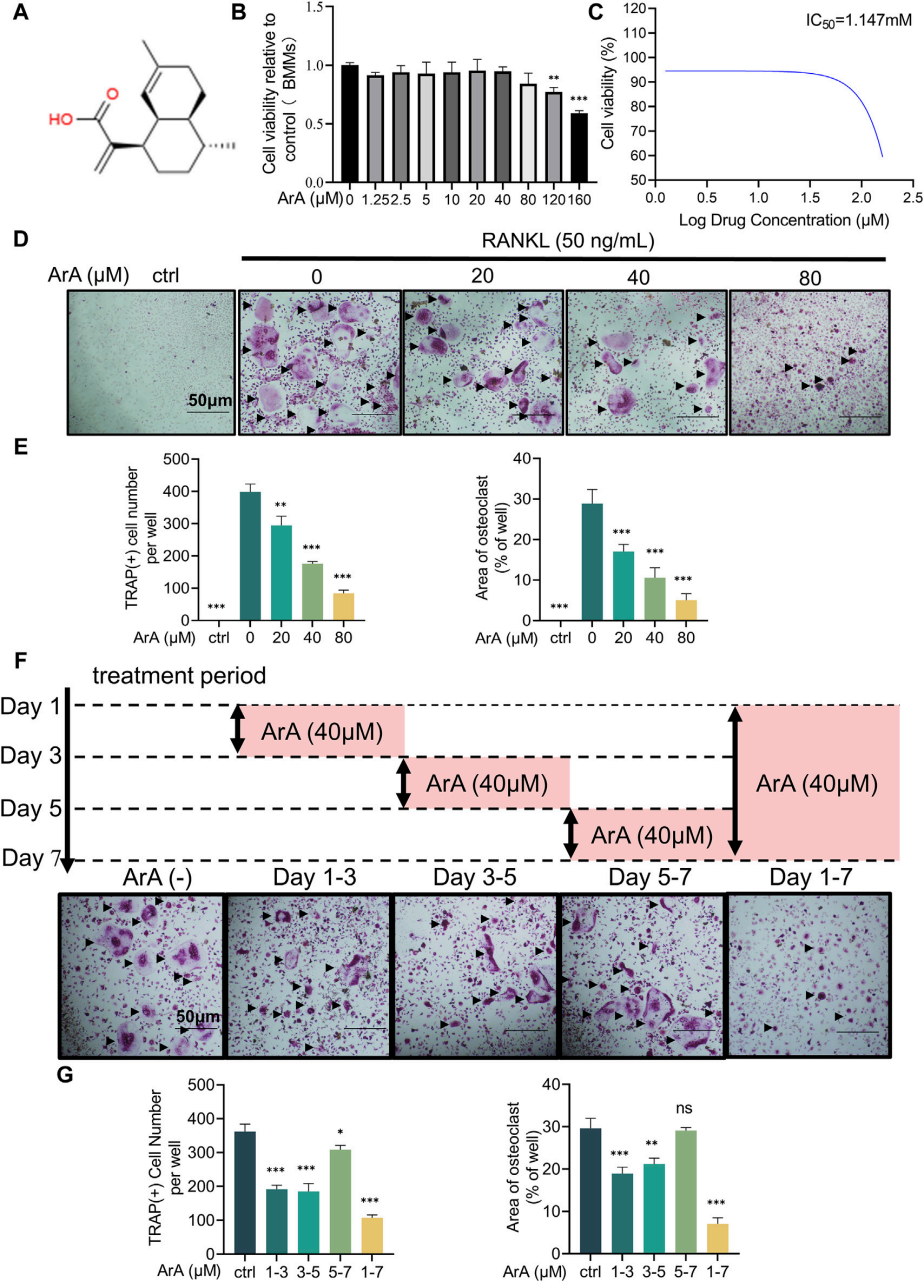
Biocompatibility and inhibitory effects of ArA on osteoclastogenesis. **(A)** Chemical structure of ArA (C15H22O2, ≥99.73% purity). **(B)** CCK-8 assay results for the cytotoxicity of ArA on BMMs. **(C)** Quantitative calculation of the IC50 of ArA in BMMs. **(D)** Representative TRAP staining images of BMMs stimulated with RANKL for 7 days and treated with various concentrations of ArA. **(E)** Quantitative analysis of the number of TRAP-positive cells and the osteoclast area per well. **(F)** Representative TRAP staining images of BMMs stimulated with RANKL for 7 days and treated with 40 μM ArA for different periods. **(G)** Quantitative analysis of the number of TRAP-positive cells and the osteoclast area per well. Data are presented as mean ± SD (*p < 0.05, **p < 0.01, ***p < 0.001 versus RANKL alone).

To assess the effect of ArA on RANKL-induced osteoclastogenesis, osteoclast precursor BMMs were incubated with two cytokines necessary for osteoclast stimulation, namely, M-CSF and RANKL, and different concentrations of ArA. A CCK-8 assay was used to test the non-cytotoxic dose range of ArA on BMMs. The results showed that ArA at concentrations ≤80 μM did not impair BMM cell viability (Figures 1A, B), demonstrating that ArA had relatively low cytotoxicity and suggesting that the inhibitory effects of ArA on osteoclast differentiation and function were not caused by cytotoxicity. The calculated IC50 value was 1.147 mM (Figure 1C). Therefore, in subsequen*t in vitro* experiments, different non-cytotoxic concentrations of ArA were used to observe their effect on multinucleate osteoclasts. TRAP staining revealed that ArA inhibited osteoclast abundance and size in a concentration-dependent manner (Figures 1D, E). Compared with that in the RANKL-only group, the formation of osteoclasts in the 20 μM ArA treatment group was reduced by approximately 30%, and osteoclast area was reduced by approximately 50%. In the 80 μM ArA treatment group, these two values increased to approximately 75%. The results showed that ArA mainly exerted an inhibitory effect in the first 4 days of osteoclast formation and had no obvious inhibitory effect during days 5–7 (Figures 1F, G). In summary, ArA inhibited RANKL-induced osteoclast formation in a dose- and time-dependent manner *in vitro*.

### 3.2 ArA inhibited F-actin ring formation and osteoclastic resorption

**FIGURE 2:**
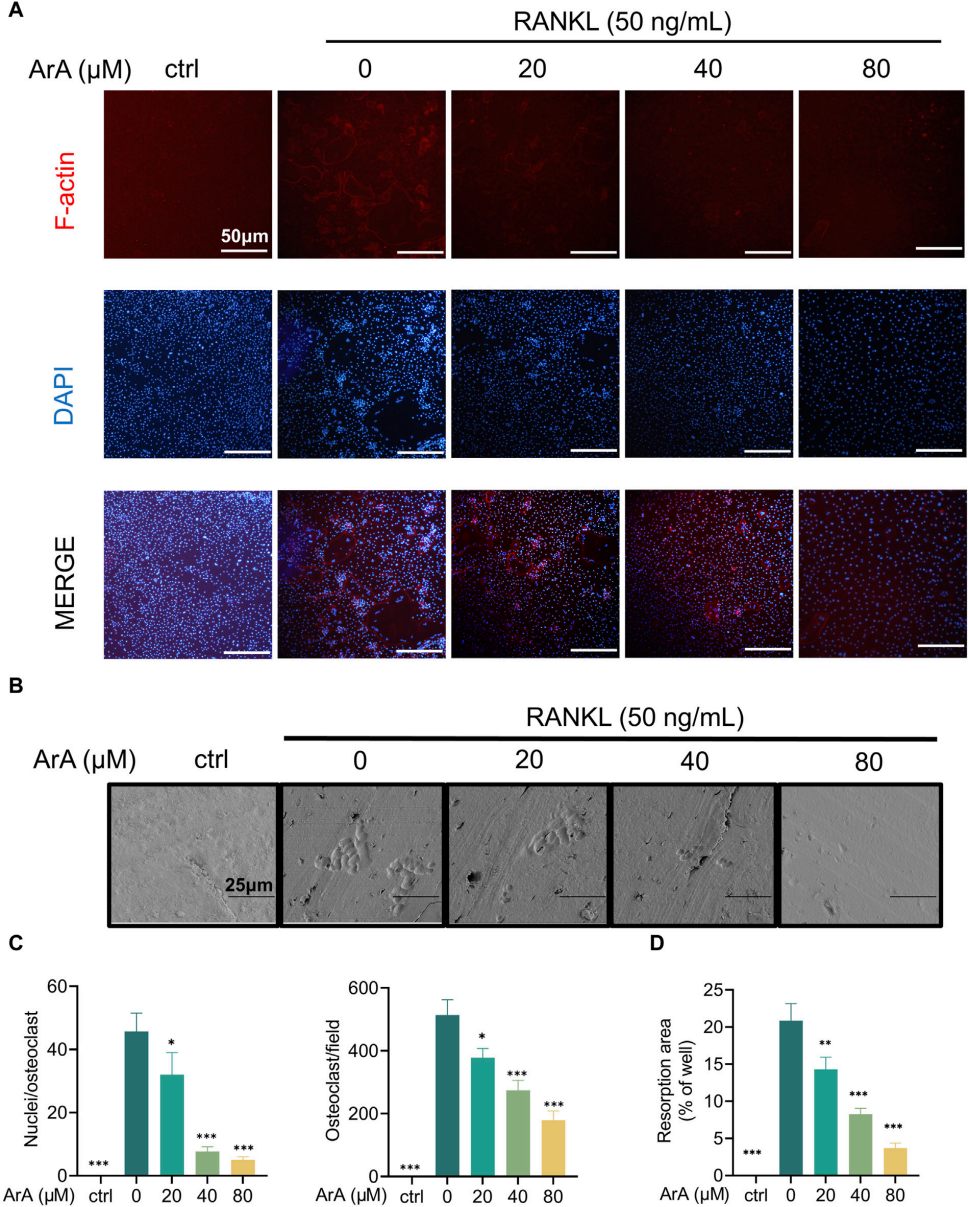
ArA inhibited F-actin ring formation and osteoclastic resorption. **(A)** Representative immunofluorescence images of F-actin rings in BMMs stimulated with RANKL for 5 days in the presence of various concentrations of ArA. Nuclei were labeled blue with DAPI and F-actin was labeled red with phalloidin tracker. **(B)** Representative images of bone resorption pits on bovine bone slices produced by RANKL-induced osteoclasts treated with various concentrations of ArA. **(C)** Ratio of nuclei/osteoclasts and number of F-actin rings per well. **(D)** Quantitative analysis of absorption pit area percentage. Data are presented as mean ± SD (*p < 0.05, **p < 0.01, ***p < 0.001 versus RANKL alone).

The adhesion of osteoclasts to the bone surface is crucial for their bone resorption function. The formation of dense, ribbon-like F-actin structures is one of the changes in mature osteoclast polarization and morphology (Teitelbaum, 2000; Vaananen et al., 2000) and plays an essential part in the resorption function of osteoclasts (Garbe et al., 2012). Therefore, we examined whether ArA affected F-actin ring formation using immunofluorescence analysis. ArA dose-dependently inhibited osteoclasts from forming intact F-actin rings, which was not observed in the control group treated with RANKL only (Figure 2A). The quantity of osteoclasts and their nuclei was reduced after ArA treatment (Figure 2B). Consistent results were observed for the erosion of bovine bone slices; scanning electron microscopy images (Figure 2C) showed that the area of resorption pits decreased as the concentration of ArA increased (Figure 2D). Therefore, ArA inhibited osteoclast F-actin formation and bone resorption function in a concentration-dependent manner.

### 3.3 ArA downregulated osteoclast-related gene expression and downstream transcription factors

**FIGURE 3:**
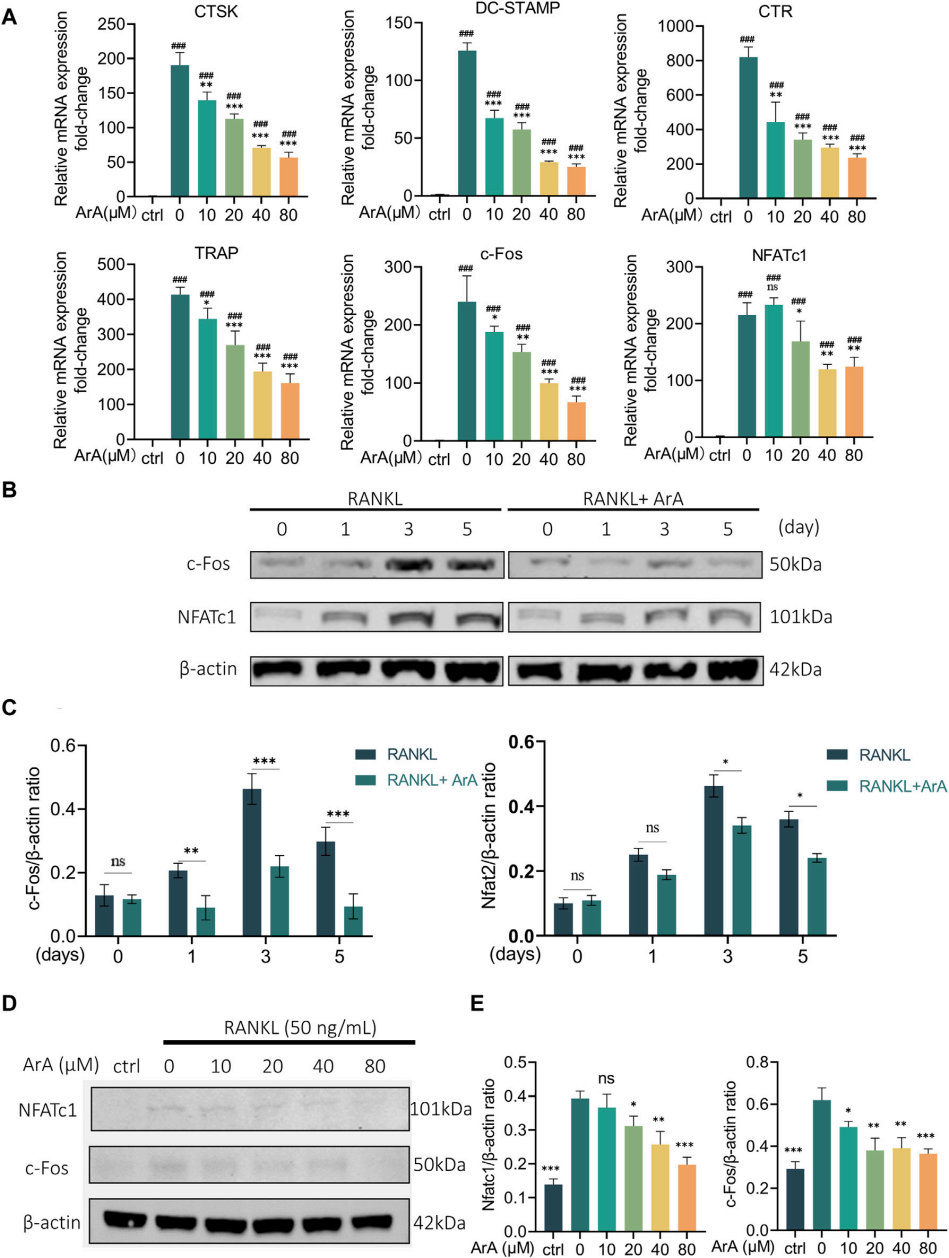
ArA inhibits RANKL-stimulated transcription and expression of osteoclast-related genes in BMMs. **(A)** RT-qPCR results of osteoclast-related gene expression in BMMs cultured with RANKL for 3 days in the presence of various ArA concentrations, with gene expression displayed as relative expression compared to the control. **(B)** Western blotting results for c-Fos and NFATc1 protein expression in BMMs treated with RANKL and 40 μM ArA at days 0, 1, 3, and 5. **(C)** Ratios of c-Fos/β-actin and NFATc1/β-actin (ArA treatment concentration: 40 μM) with β-actin as internal reference protein. **(D)** Western blotting results for c-Fos and NFATc1 protein expression in BMMs stimulated by RANKL for 3 days in the presence of different concentrations of ArA with β-actin as internal reference protein. **(E)** Ratios of c-Fos/β-actin and NFATc1/β-actin. Data are presented as mean ± SD (ns, not significant, *p < 0.05, **p < 0.01, ***p < 0.001 versus RANKL alone; ^###^ *p* < 0.001 compared with control group).

RT-qPCR was used to further explore the effect of ArA on formation- and function-related gene transcription in RANKL-induced osteoclasts at the mRNA level. As shown in Figure 3A, stimulation of RANKL caused BMMs to differentiate into osteoclasts. The expression of osteoclast-related genes in each group induced by RANKL was significantly increased compared with the control group (*p* < 0.001). The mRNA transcript levels of osteoclast formation- and function-related genes in the ArA-treated group were lower than those with only RANKL stimulation. TRAP and CTR are closely related to differentiation, whereas DC-STAMP and CTSK are related to osteoclast fusion and bone resorption, respectively (Garbe et al., 2012). c-Fos and NFATc1 are the key downstream transcription factors that initiate osteoclast differentiation (Gohda et al., 2005). In the control RANKL-stimulated osteoclasts, protein levels of NFATc1 and c-Fos were enhanced, reaching the highest level on day 3 and then decreasing slightly by day 5. Protein expression at these two time points was inhibited by ArA (Figures 3B, C). To further explore the effects of ArA, BMMs were treated with different concentrations of ArA. The protein levels of NFATc1 and c-Fos were reduced upon ArA treatment, and as the concentration increased, the inhibition became more significant (Figures 3D, E). To investigate the effects of ArA on osteoclast formation at different stages, we have included experiments using RAW264.7 murine macrophages alongside BMMs. We verified by the CCK-8 kit that ArA has no cytotoxicity to RAW264.7 murine macrophages in the concentration range of 0–320 μM. Then we stimulated RAW264.7 murine macrophages with RANKL and co-cultured them with different concentrations of ArA for 6 days, and we detected the levels of osteoclast-related transcription factors by RT-qPCR. The results are shown in Supplementary Figure S2, which, together with the BMMs experimental results, indicate that ArA not only has a significant effect on inhibiting the differentiation of BMMs into osteoclasts but also inhibits the differentiation of RAW264.7 murine macrophages into osteoclasts. Therefore, ArA suppressed the expression of osteoclast formation- and function-related genes and proteins in a time- and concentration-dependent manner.

### 3.4 ArA does not affect osteoblast differentiation and mineralization

Bone homeostasis is a complex process that requires the coordinated actions of osteoblasts and osteoclasts (Simon et al., 2017). Therefore, in addition to the effect on osteoclast differentiation and bone resorption function, it is also important to study the effect of ArA on osteoblast differentiation and mineralization. First, a CCK-8 assay was performed to assess the cytotoxicity of ArA on BMSCs (Supplementary Figure S3A). The results showed that ArA at concentrations ≤160 μM did not impair BMSC cell viability. To explore the effect of ArA on osteoblastogenesis and mineralization, BMSCs were cultured in osteogenic induction medium with different concentrations of ArA (0, 40, and 80 μM). Cells were stained with ALP and alizarin red after 7 and 21 days of culture, respectively (Supplementary Figures S3B, C). The results indicated that ArA had no significant effect on osteoblast differentiation and mineralization.

### 3.5 ArA attenuated RANKL-induced MAPK and NF-κB pathway activation

**FIGURE 4:**
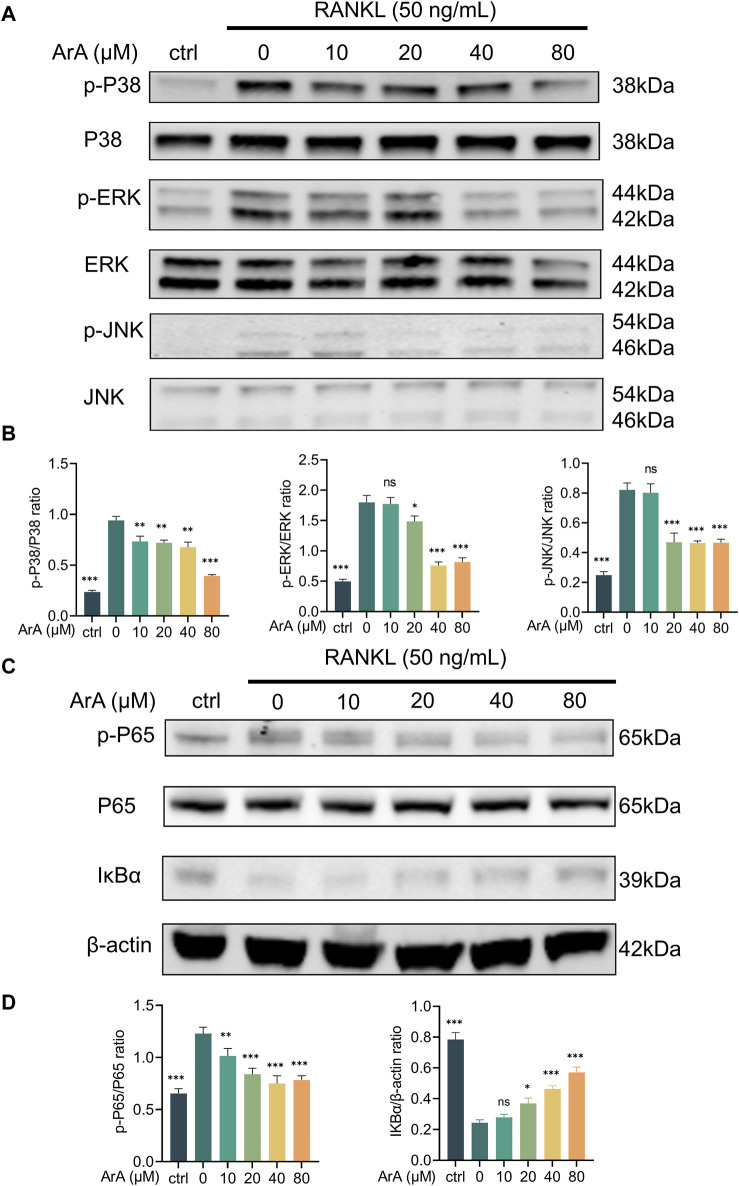
ArA suppresses RANKL-induced osteoclast differentiation via the NF-κB and MAPK pathways. **(A)** Western blotting results of MAPK-family protein expression in BMMs stimulated by RANKL for 25 min in the presence of different concentrations of ArA. **(B)** Ratios of p-p38/p-38, p-ERK/ERK, and p-JNK/JNK in the presence of different concentrations of ArA. **(C)** Western blotting results of NF-κB pathway protein expression in BMMs stimulated by RANKL for 25 min in the presence of different concentrations of ArA. **(D)** Ratios of p-p65/p65 and IκBα/β-actin in the presence of different concentrations of ArA with β-actin as internal reference protein. The data are presented as the mean ± SD (*p < 0.05, **p < 0.01, ***p < 0.001 versus RANKL alone).

Western blotting was used to study the role of the NF-κB and MAPK pathways, two important osteoclastogenic signaling pathways upstream of NFATc1 and c-Fos, respectively, in ArA’s inhibitory effect on osteoclast differentiation and function. The results showed that the phosphorylation levels of MAPK pathway elements (i.e., ERK, JNK, and p38) were significantly upregulated after RANKL stimulation for 25 min (Figures 4A, B). This upregulation was inhibited by ArA treatment in a concentration-dependent manner. Specifically, the phosphorylated protein ratio in the 80 μM ArA treatment group was approximately 50% that in the control group. Similar results were observed for the phosphorylation of p65 in the NF-κB pathway. Furthermore, ArA treatment visibly inhibited the RANKL-induced degradation of IκBα (Figures 4C, D), which is an inhibitor of NF-κB. Therefore, ArA inhibited RANKL-induced MAPK and NF-κB pathway activation in a dose-dependent manner.

### 3.6 ArA suppressed RANKL-induced ROS accumulation by activating the Nrf2 pathway

**FIGURE 5:**
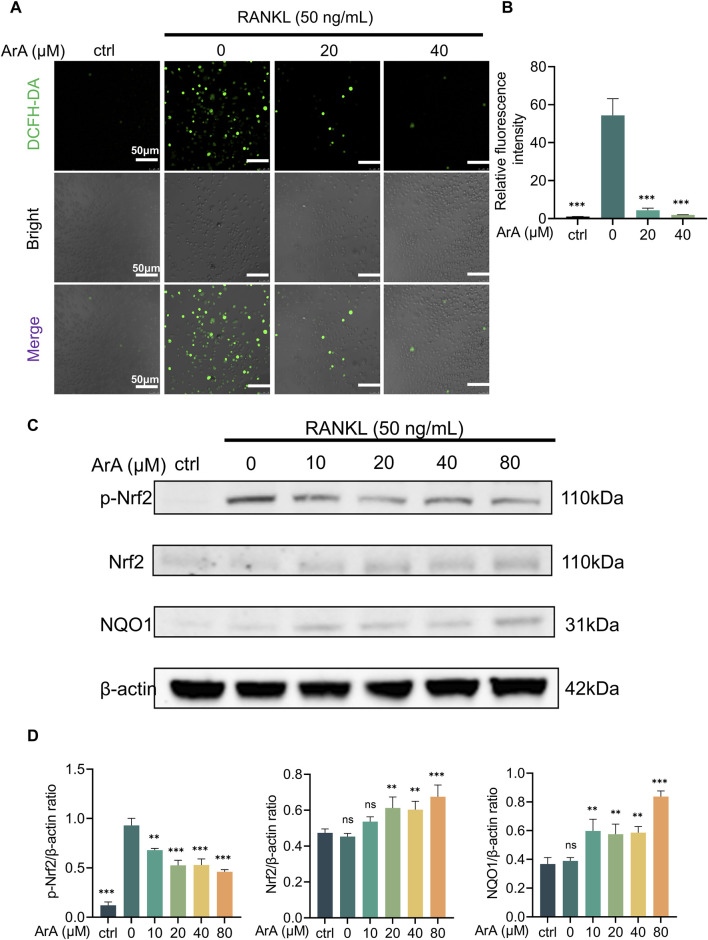
ArA suppressed RANKL-induced ROS production by activating the Nrf2 pathway. **(A)** Representative immunofluorescence images of intracellular ROS in BMMs stimulated with RANKL for 24 h and treated with different concentrations of ArA. DCFH labels intracellular ROS in green. **(B)** Quantitative analysis of relative fluorescence intensity. **(C)** Western blotting results of Nrf2 pathway protein expression in BMMs stimulated by RANKL for 25 min in the presence of different concentrations of ArA. **(D)** Ratios of p-Nrf2/β-actin, Nrf2/β-actin, and NQO1/β-actin in BMMs stimulated by RANKL for 25 min in the presence of different concentrations of ArA. The data are presented as the mean ± SD (**p < 0.01, ***p < 0.001 versus RANKL alone).

Considering that increased intracellular ROS levels are indispensable for osteoclastogenesis (Lee et al., 2005) and that ArA reportedly has antioxidant effects (Ferreira and Luthria, 2010), we further explored the effect of ArA on intracellular ROS levels during osteoclast formation using a DCFH-DA probe. Intracellular ROS levels were significantly increased by RANKL stimulation. The relative fluorescence intensity showed that ArA treatment significantly suppressed this effect, with the ROS level in the 40 μM treatment group suppressed to less than 5% of its normal level (Figures 5A, B). To further investigate the molecular mechanism by which ArA inhibits intracellular ROS levels, Western blotting was used to study the Nrf2 signaling pathway, which is closely related to the cellular restriction of ROS production. The results demonstrated that RANKL stimulation-induced Nrf2 phosphorylation was attenuated by ArA treatment. In addition, ArA increased the levels of Nrf2 proteins and its downstream target, NQO1 (Figure 5C). Our analysis indicated that RANKL stimulation did not significantly impact the protein expression of Nrf2 or NQO1, whereas treatment with 20 μM ArA increased Nrf2 and NQO1 by approximately 40% (Figure 5D). Therefore, ArA suppressed intracellular ROS levels in cells undergoing osteoclastogenesis by activating Nrf2 and downstream NQO1 dose-dependently.

### 3.7 ArA attenuated Ti particle-induced calvarial osteolysis *in vivo*

**FIGURE 6:**
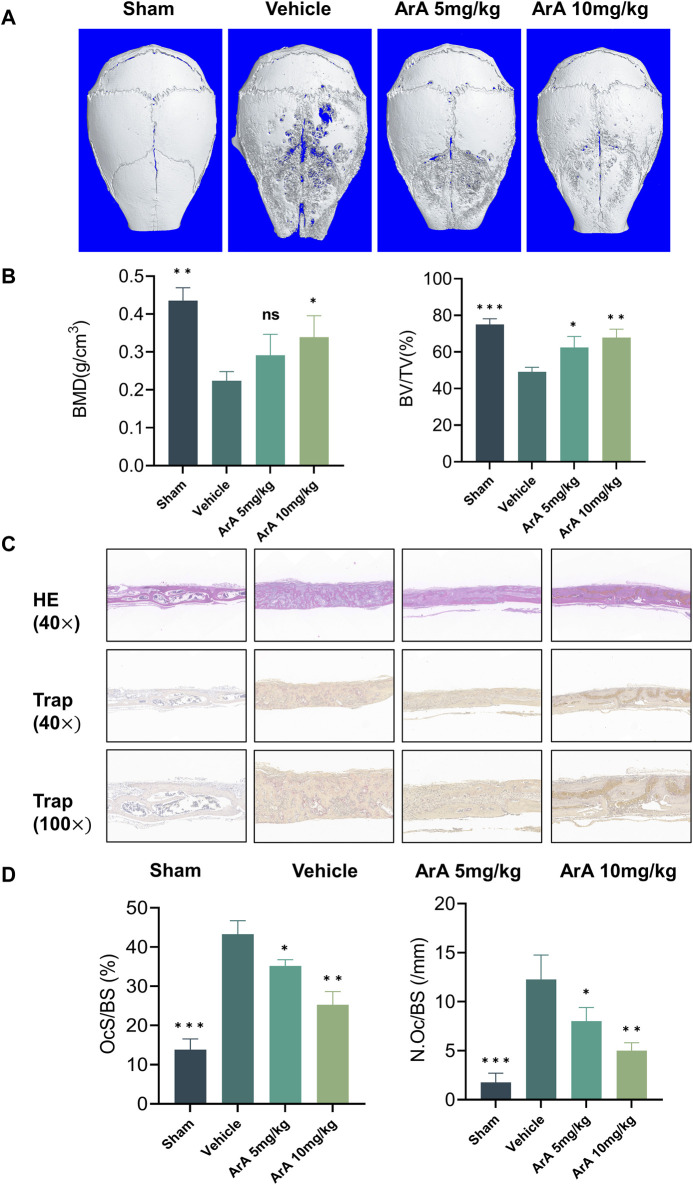
ArA inhibited Ti particle-induced calvarial osteolysis *in vivo*. **(A)** Representative micro-CT scanning images of mice calva. **(B)** Quantitative analysis of BV/TV and BMD as measured from micro-CT scanning images. **(C)** Representative images of H&E and TRAP staining indicating the decreased osteolytic region and TRAP-positive osteoclasts in the ArA-treated groups. **(D)** Quantitative analysis of N. Oc/BS and OcS/BS ratio by histological staining. The data are presented as mean ± SD (*p < 0.05, **p < 0.01, ***p < 0.001 versus the vehicle group).

Based on the above *in vitro* experiments showing that ArA inhibits osteoclast formation, we constructed a mouse Ti particle-induced calvarial osteolysis model to investigate the effect of ArA on osteoclast-associated osteolysis *in vivo* and explore its potential applications in treating PPO. Micro-CT results indicated that the Ti particles induced extensive bone erosion on the calvarial surface, with large, deep pits in the vehicle-treated group. However, ArA treatment rescued osteolysis in a concentration-dependent manner, as the extent of bone erosion in the 5 mg/kg ArA treatment group was visibly lower than that in the vehicle group, and this effect was even more significant in the 10 mg/kg ArA treatment group (Figure 6A). Quantitative analysis of bone parameters further assured the effect of ArA. Specifically, the BMD and BV/TV of the ArA-treated group were significantly increased compared with those of the vehicle group (Figure 6B).

**FIGURE 7:**
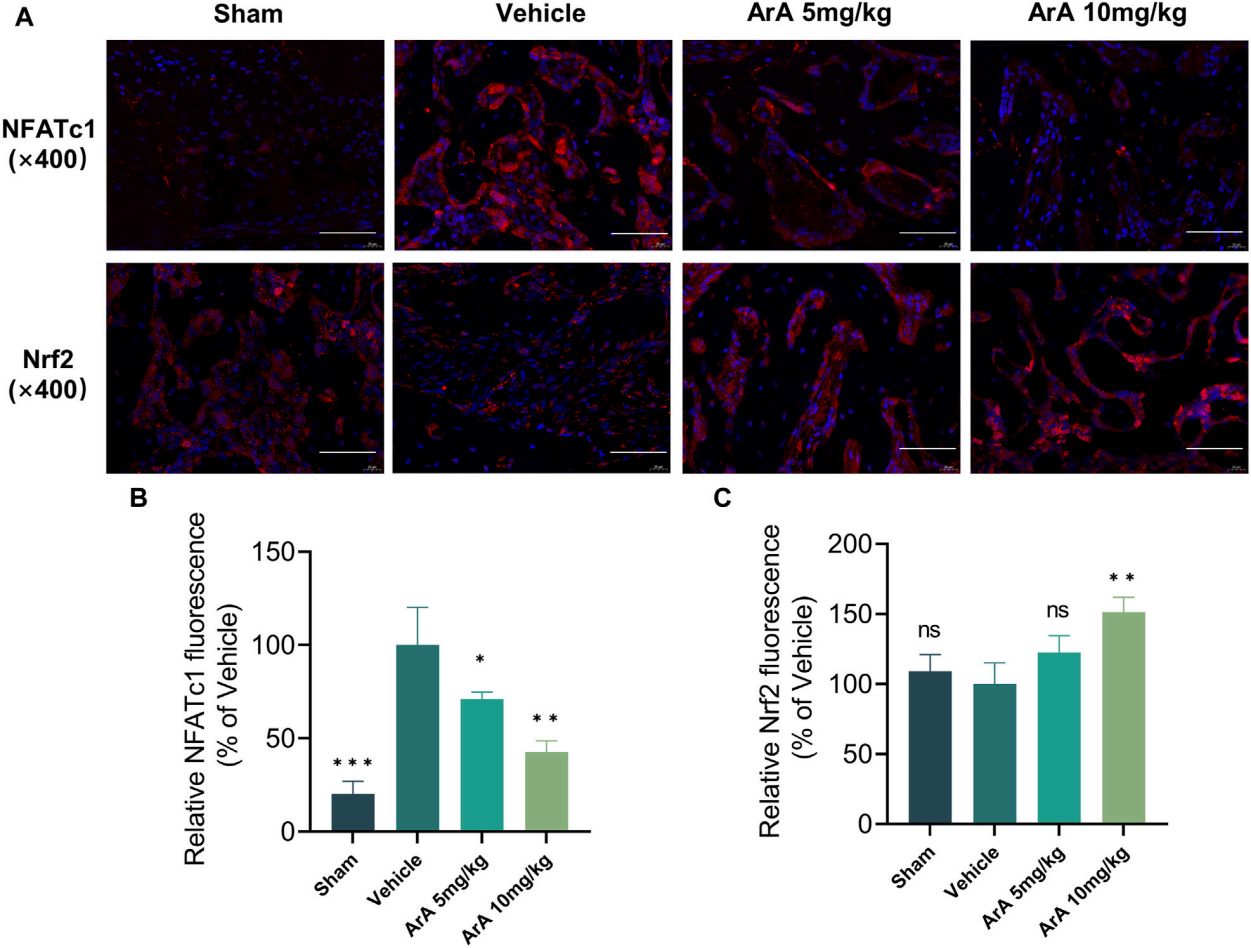
Inhibitory effect of ArA on Ti-induced calvarial osteolysis. **(A)** Representative immunofluorescence images of NFATc1 and Nrf2. **(B)** Quantitative analysis of relative NFATc1 fluorescence. **(C)** Quantitative analysis of relative Nrf2 fluorescence. Data are presented as mean ± SD (ns, not significant, **p < 0.01, ***p < 0.001 versus the vehicle group).

The results of the histomorphological evaluation were consistent with those of the micro-CT analysis, further confirming that ArA inhibited Ti particle-induced osteolysis. Specifically, H&E staining showed that localized Ti particles caused extensive osteolysis, whereas ArA alleviated this effect (Figure 6C). TRAP staining showed that TRAP^+^ osteoclasts in the vehicle group were abundant and widely distributed, and clear bone resorption was observed. In the ArA-treated group, the number of multinucleated TRAP^+^ cells and the OcS/BS ratio were both significantly reduced (Figure 6D), as was the degree of osteolysis. Immunofluorescence analysis was used to assess the molecular mechanisms of the ArA effect *in vivo*. Figure 7 shows that compared with the sham operation group, the NFATc1 level in the Vehicle group was significantly increased (*p* < 0.001), while there was no significant difference in Nrf2 levels between the two groups. Compared with the vehicle group, the NFATc1 level in the ArA treatment group decreased significantly (*p* < 0.05), and the 10 mg/kg ArA treatment group decreased more significantly (*p* < 0.01). Moreover, we tested the biosafety of ArA *in vivo*. H&E staining revealed no obvious adverse effects on the histological structures of major organs, including the heart, liver, and kidneys, as shown in Supplementary Figure S4. Overall, immunohistochemical results further demonstrated the concentration-dependent protective effects of ArA on Ti particle-induced calvarial osteolysis.

## 4 Discussion

**FIGURE 8:**
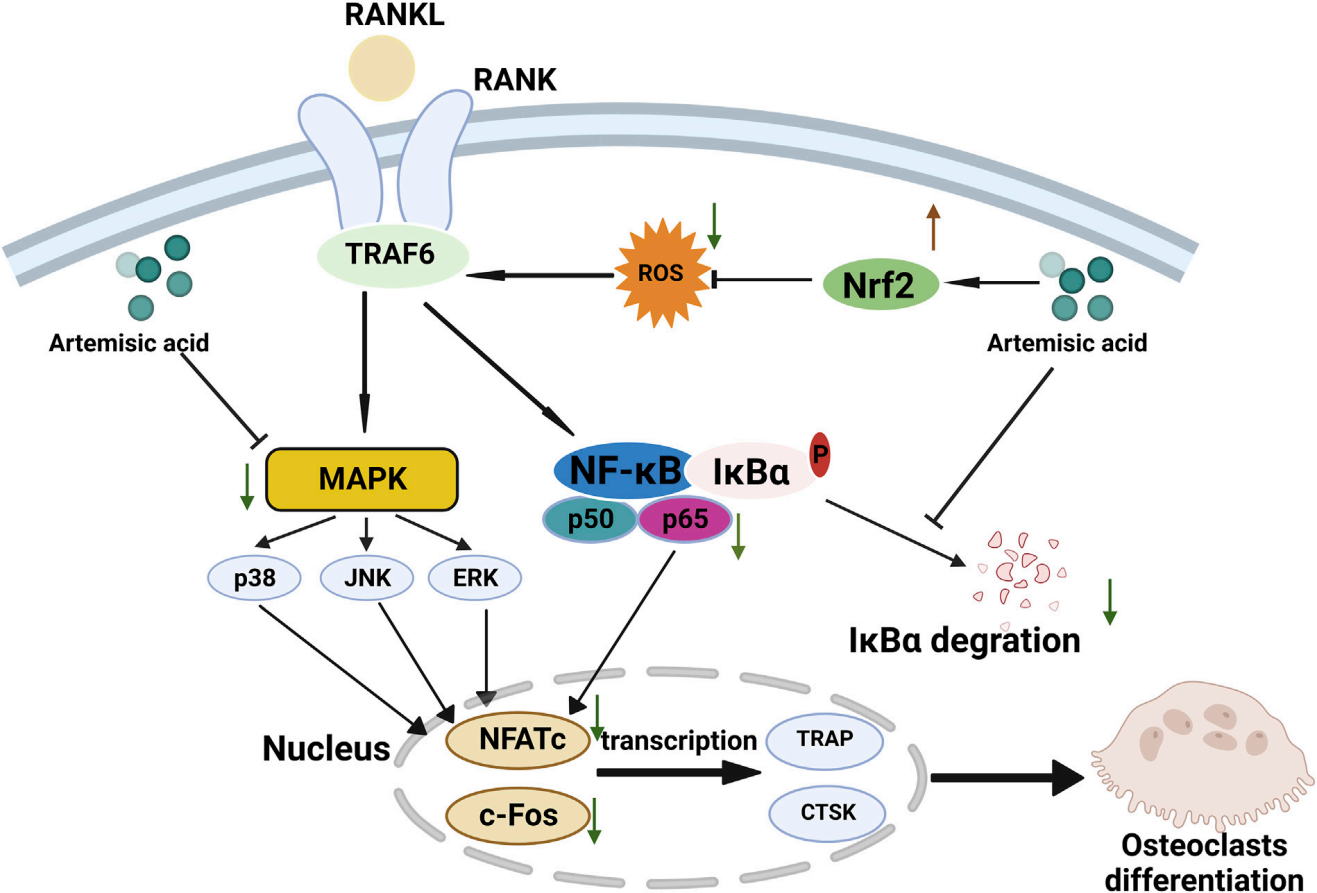
Proposed scheme for inhibitory mechanism of ArA on RANKL-induced osteoclastogenesis. RANKL binds with RANK, induces ROS generation, activates downstream MAPK and NF-κB pathways, increases the expression of NFATc1 and c-Fos, and upregulates the transcription of osteoclast-specific genes such as Ctsk and Trap, ultimately leading to osteoclast differentiation. ArA induces an osteoprotective effect by upregulating Nrf2 expression to suppress intracellular ROS levels while downregulating MAPK and NF-κB pathways.

Here, we explored the possible osteoprotective effects of ArA and found that it attenuated RANKL-induced osteoclastogenesis and bone resorption *in vitro* and attenuated Ti particle-induced bone erosion *in vivo*. The underlying mechanism involved upregulating Nrf2 expression to suppress intracellular ROS accumulation and downregulating RANKL-stimulated MAPK and NF-κB pathway activation during the process of osteoclast differentiation and function (Figure 8). ArA is a natural sesquiterpene isolated from *Artemisia annua* L. that has various biological activities, including antioxidant and antimalarial effects. The pharmacological effects of artemisinic acid on osteoclast differentiation and the treatment of osteolysis have not yet been explored. Our work demonstrated the relatively low cytotoxicity of ArA in BMMs *in vitro*, which supports the conclusions of Zheng concerning the relative cytotoxicity of ArA in other cell lines (Zheng, 1994). Other studies have explored the inhibitory effect of artesunate on osteoclast formation and bone loss (Wei et al., 2018) and the inhibitory effect of the ethanol extract of *Artemisia annua* L., which contains multiple active ingredients, on osteoclastogenesis and osteoporosis (Lee et al., 2017). To the best of our knowledge, this is the first study to investigate the osteoprotective effects of ArA on osteoclast formation and function as well as Ti particle-induced osteolysis.

In this study, we found that ArA inhibits RANKL-stimulated osteoclast differentiation and bone resorption *in vitro*, as confirmed by osteoclast characteristic staining with TRAP, immunofluorescence staining for F-actin, bone resorption assays, RT-qPCR, and Western blotting. Meanwhile, ALP and alizarin red staining jointly demonstrated that ArA had no obvious effect on osteoblast differentiation and mineralization. The molecular mechanism inhibits ROS by upregulating the Nrf2 pathway and downregulating MAPK and NF-κB signaling pathways, thereby inhibiting the transcription and expression of characteristic genes, including key transcription factors c-Fos and NFATc1. Consistently, ArA exhibited a significant inhibitory effect on Ti-induced calvarial osteolysis, as confirmed by micro-CT, immunohistochemistry staining of H&E, and TRAP.

We then explored the mechanism of action of ArA. RANKL binds with the receptor RANK, and adapters, including TRAF6, are recruited, subsequently upregulating NF-κB and MAPK pathways and elevating ROS levels (Agidigbi and Kim, 2019), jointly inducing the transcription of c-Fos and NFATc1, regulating osteoclast differentiation and function. NFATc1 is an important initiating transcription factor that can be activated by NF-κB signaling, MAPK signaling, c-Fos, and Ca^2+^. c-Fos and NFATc1 jointly regulate the transcription of characteristic osteoclastic cytokines, including TRAP and CTSK. We reported that ArA can significantly suppress osteoclast formation by downregulating RANKL-induced MAPK and NF-κB pathway activation and reducing the transcription of NFATc1 and c-Fos in a time- and concentration-dependent manner, thereby inhibiting the transcription of osteoclast-related genes. In addition, RANKL-induced ROS production is another mechanism that underlies osteoclast differentiation (Ha et al., 2004). Under oxidative stress, Nrf2 is separated from Keap1, and Nrf2 regulates the expression of NQO1, an antioxidant factor. We found that ArA reduced osteoclast formation by increasing free Nrf2 and upregulating the expression of the antioxidase NQO1, thereby reducing intracellular ROS levels. Based on *in vitro* results, we then established a Ti particle-induced mouse calvarial osteolysis model to further investigate the *in vivo* potential effects of ArA. Micro-CT imaging showed significant bone erosion in the vehicle group. In contrast, ArA treatment increased the BV/TV ratio and BMD. H&E and TRAP staining also demonstrated that ArA reduced erosion and lowered the quantity of osteoclasts in a concentration-dependent manner.

This study had some limitations. First, we reported that ArA can alleviate RANKL-induced elevation of intracellular ROS levels, which we speculated was a possible mechanism by which ArA inhibits osteoclastogenesis. Meanwhile, cytokines secreted by osteoblasts are crucial for osteoclastogenesis. In the inflammatory response of pathophysiological processes such as PPO, intracellular ROS in osteoblast precursor cells and osteoblasts increases, leading to cell death (Sun et al., 2021). Therefore, the effects of ArA on osteoblast death caused by the increase in intracellular ROS under inflammatory stimulation still need to be further explored. Second, in actual PPO situations, titanium particles are not the only wear particles present (Goodman, 2007). Therefore, the established results may not be replicated under actual PPO conditions, and the effect of ArA on osteolysis induced by other particles remains to be explored. Third, the pathogenesis of wear particle-induced osteoclastogenesis involves inflammatory responses (Purdue et al., 2007), and the effects of ArA on these processes are unclear. Therefore, studies are required to gain a deeper insight into the effect and mechanism of ArA in condition of inflammatory response to wear particles.

According to current research on drug treatment of Ti particle-induced mouse cranium osteolysis model (Qu et al., 2019; Yamada et al., 2021; Niu et al., 2024) and the administration of the artemisinin family in mice (Wu et al., 2018; Liang et al., 2023), intraperitoneal treatment is the preferable route of administration that primarily hinges on its high bioavailability and direct delivery to the systemic circulation it provides, bypassing the gastrointestinal tract and the first-pass effect in the liver. Moreover, the precise control over dosage and timing that intraperitoneal administration offers is critical in experimental settings. Nonetheless, previous studies have demonstrated the potential for oral use of artemisinic acid (Jung et al., 2002; Fu et al., 2021), which may also be the direction for further research.

In summary, our study demonstrated that ArA significantly alleviated bone corrosion in a Ti particle-induced mouse cranium osteolysis model and attenuated osteoclast differentiation and function *in vitro*, possibly by upregulating Nrf2 to inhibit ROS levels while downregulating MAPK and NF-κB pathways and their downstream factors. The highlight of this study is that we report the bone-protective function of ArA and further verify the pathway that inhibits osteoclasts for the first time, providing ideas for the use of small molecules derived from Chinese herbal medicine for osteoclast-related diseases. Therefore, we propose that ArA has promising potential for further development of drugs for the treatment or prophylaxis of osteoclast-associated osteolytic diseases, including PPO, arthritis, and postmenopausal osteoporosis.

## Data Availability

The datasets presented in this study can be found in online repositories. The names of the repository/repositories and accession number(s) can be found in the article/Supplementary Material.
