# Supplementary material for: Artemisinic acid attenuates osteoclast formation and titanium particle-induced osteolysis via inhibition of RANKL-induced ROS accumulation and MAPK and NF-κB signaling pathways
Source: Front Pharmacol. 2024 May 1;15:1345380. doi: 10.3389/fphar.2024.1345380 (PMC11094322; doi:10.3389/fphar.2024.1345380)
Supplement: Supplementary file 1 [file DataSheet1.docx]

Supplementary Material

Artemisinic acid attenuates osteoclast formation and titanium particle-induced osteolysis via inhibition of RANKL-induced ROS accumulation and MAPK and NF-κB signaling pathways

**Tian Gao, Chaohong Yu, Xiaofeng Shi, Hu Yuehao, Yongyun Chang, Jingwei Zhang, Yitian Wang, Zanjing Zhai, Xinlin Jia and Yuanqing Mao***

*** Correspondence:** Yuanqing Mao: yuanqingmao@163.com

Supplementary Figures


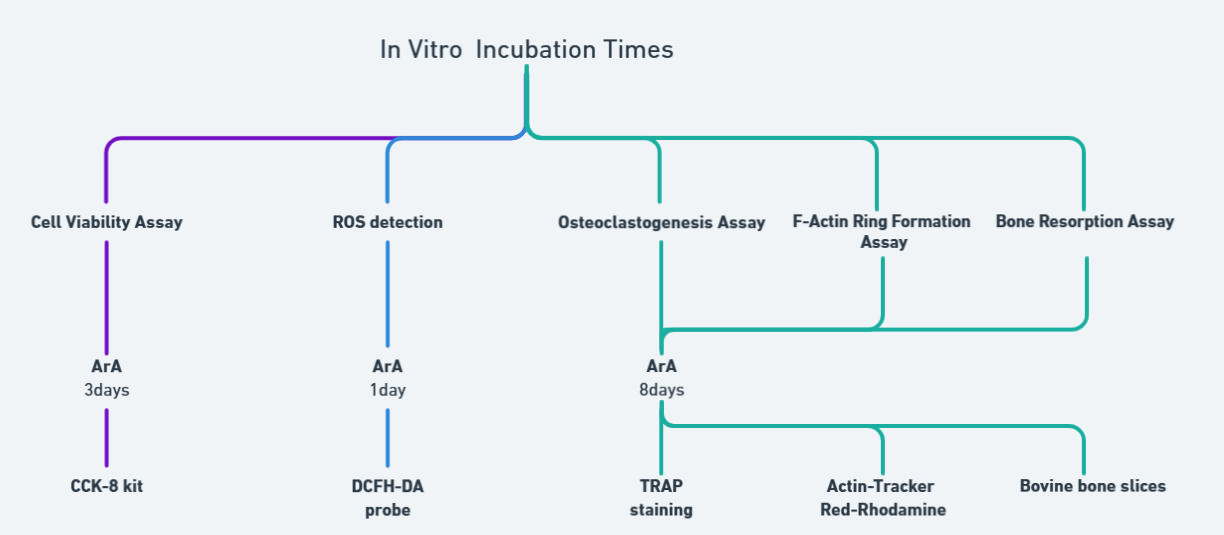


**Supplementary Figure S1** Schematic designing regarding the different experimental designs used.


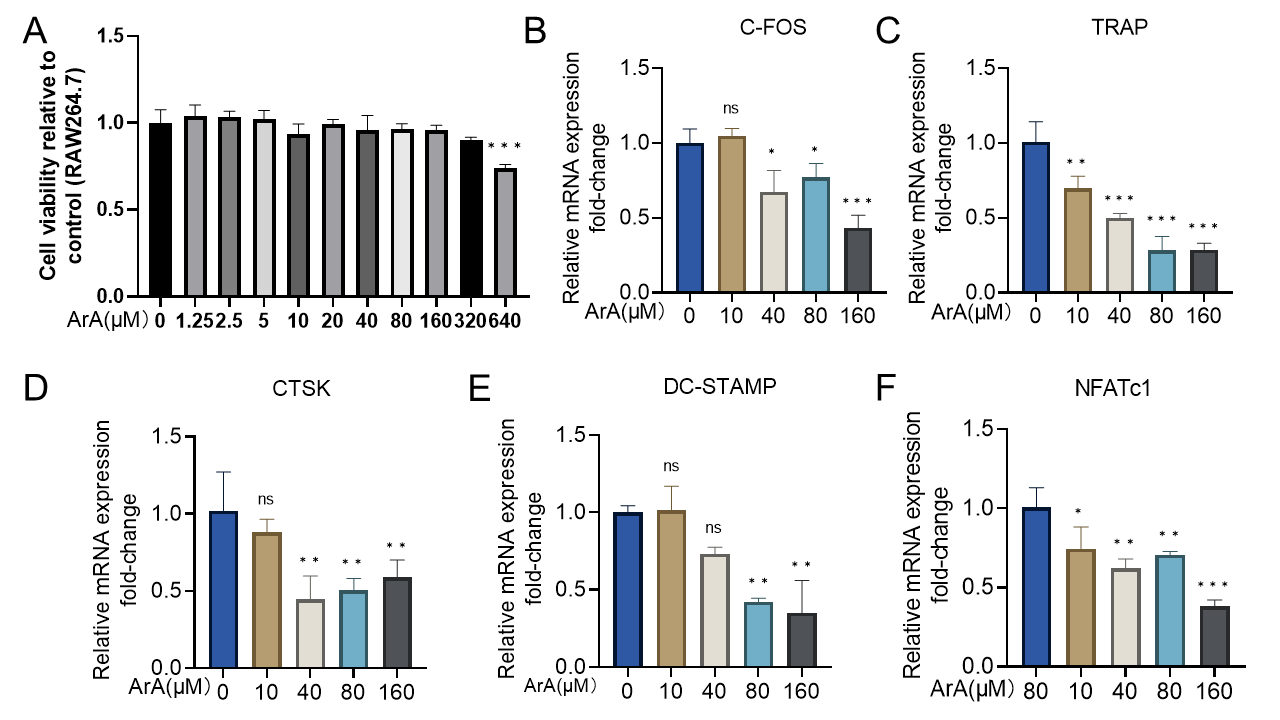


**Supplementary Figure S2.** Cytotoxicity of ArA to RAW264.7 and inhibition of its osteoclast differentiation. (A) CCK-8 assay results for the cytotoxicity of ArA on RAW264.7. (B-F) RT-qPCR results of osteoclast-related gene expression in RAW264.7 cultured with RANKL for 6 d in the presence of various ArA concentrations. Data are presented as mean ± SD (ns - not significant, *p < 0.05, **p < 0.01, ***p < 0.001 versus RANKL alone).


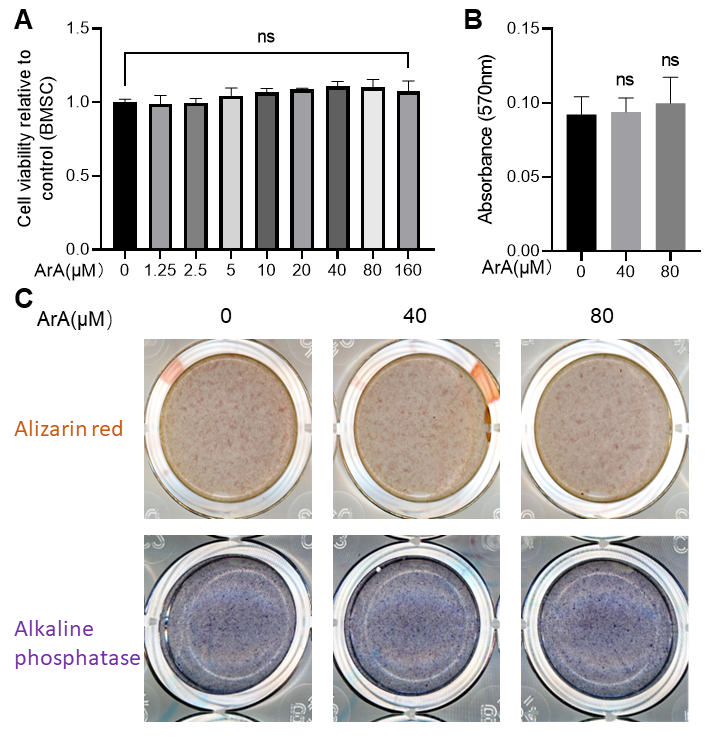


**Supplementary Figure S3.** Effects of ArA on cytotoxicity of BMSCs and their osteogenic differentiation. (A) CCK-8 assay results for the cytotoxicity of ArA on BMSCs for 72h. (B) The stained dye was extracted and measured at an absorbance of 570 nm. (C) The effect of ArA on mineralized nodule formation was analyzed by alizarin red S and ALP expression in was determined using an ALP Staining Kit. Data are presented as mean ± SD (ns - not significant).


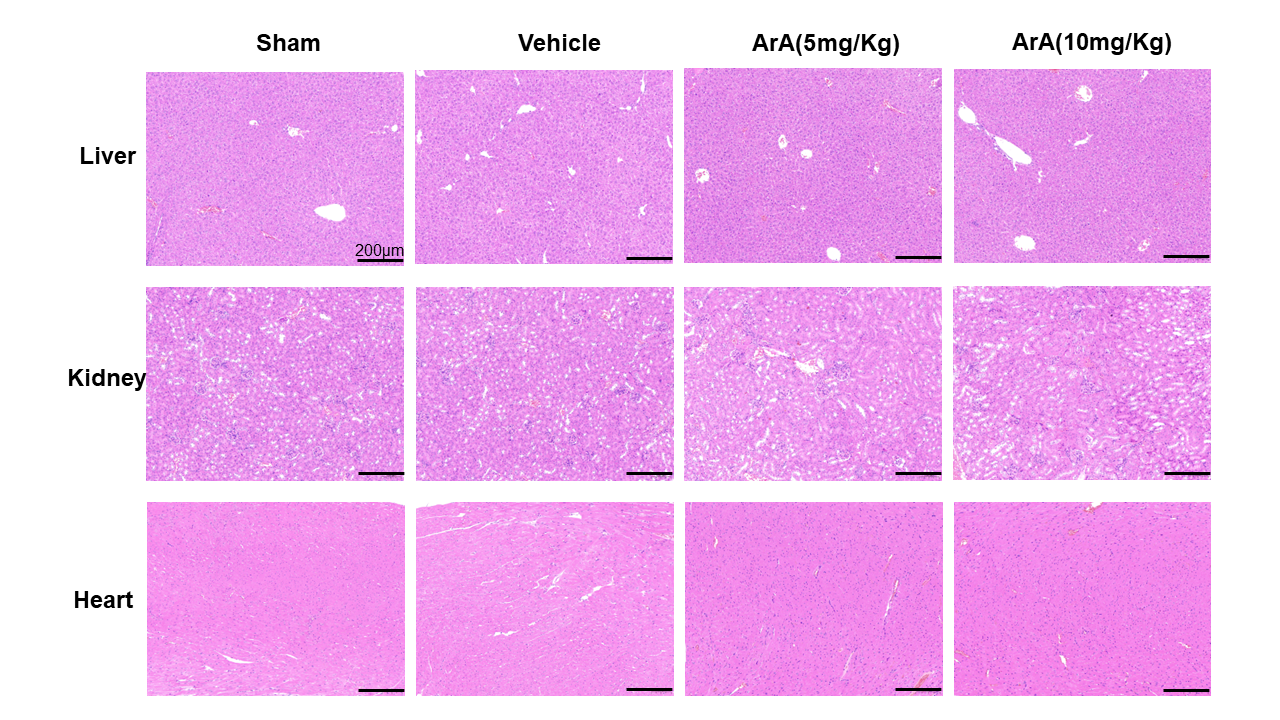


**Supplementary Figure S4.** H&E staining images of liver, kidney and heart 14 days after ArA treatment.
